# Enhancing pneumonia prognosis in the emergency department: a novel machine learning approach using complete blood count and differential leukocyte count combined with CURB-65 score

**DOI:** 10.1186/s12911-024-02523-1

**Published:** 2024-05-03

**Authors:** Yin-Ting Lin, Ko-Ming Lin, Kai-Hsiang Wu, Frank Lien

**Affiliations:** 1 Department of Internal Medicine, Chang Gung Memorial Hospital, No. 6, W. Sec., Jiapu Rd., Puzih, Chiayi County 613 Taiwan; 2 Division of Allergy, Immunology and Rheumatology, Department of Internal Medicine, Chang Gung Memorial Hospital, No. 6, W. Sec., Jiapu Rd, Puzih, Chiayi County 613 Taiwan; 3 Department of Emergency Medicine, Chang Gung Memorial Hospital, No. 6, W. Sec., Jiapu Rd., Puzih, Chiayi County 613 Taiwan; 4 Department of Nursing, Chang Gung University of Science and Technology, Chiayi Campus, Chiayi, Taiwan; 5 Graduate Institute of Clinical Medical Sciences, College of Medicine, Chang Gung University, Taoyuan, Taiwan

**Keywords:** Pneumonia, CURB-65, Machine learning, Blood count, Differential count, Blood Culture Prediction Index (BCPI) model, Cox regression model, Emergency department

## Abstract

**Background:**

Pneumonia poses a major global health challenge, necessitating accurate severity assessment tools. However, conventional scoring systems such as CURB-65 have inherent limitations. Machine learning (ML) offers a promising approach for prediction. We previously introduced the Blood Culture Prediction Index (BCPI) model, leveraging solely on complete blood count (CBC) and differential leukocyte count (DC), demonstrating its effectiveness in predicting bacteremia. Nevertheless, its potential in assessing pneumonia remains unexplored. Therefore, this study aims to compare the effectiveness of BCPI and CURB-65 in assessing pneumonia severity in an emergency department (ED) setting and develop an integrated ML model to enhance efficiency.

**Methods:**

This retrospective study was conducted at a 3400-bed tertiary medical center in Taiwan. Data from 9,352 patients with pneumonia in the ED between 2019 and 2021 were analyzed in this study. We utilized the BCPI model, which was trained on CBC/DC data, and computed CURB-65 scores for each patient to compare their prognosis prediction capabilities. Subsequently, we developed a novel Cox regression model to predict in-hospital mortality, integrating the BCPI model and CURB-65 scores, aiming to assess whether this integration enhances predictive performance.

**Results:**

The predictive performance of the BCPI model and CURB-65 score for the 30-day mortality rate in ED patients and the in-hospital mortality rate among admitted patients was comparable across all risk categories. However, the Cox regression model demonstrated an improved area under the ROC curve (AUC) of 0.713 than that of CURB-65 (0.668) for in-hospital mortality (*p*<0.001). In the lowest risk group (CURB-65=0), the Cox regression model outperformed CURB-65, with a significantly lower mortality rate (2.9% vs. 7.7%, *p*<0.001).

**Conclusions:**

The BCPI model, constructed using CBC/DC data and ML techniques, performs comparably to the widely utilized CURB-65 in predicting outcomes for patients with pneumonia in the ED. Furthermore, by integrating the CURB-65 score and BCPI model into a Cox regression model, we demonstrated improved prediction capabilities, particularly for low-risk patients. Given its simple parameters and easy training process, the Cox regression model may be a more effective prediction tool for classifying patients with pneumonia in the emergency room.

**Supplementary Information:**

The online version contains supplementary material available at 10.1186/s12911-024-02523-1.

## Background

Pneumonia is the leading cause of mortality and hospitalization globally [1–3]. Nonetheless, a precise assessment of its severity is crucial for effectively managing the condition and informing critical decisions regarding diagnosis, treatment, and healthcare intervention [4–6]. Early identification of patients at high risk of rapid pneumonia progression can facilitate prompt intervention, thereby ensuring patient safety and optimizing clinical outcomes [7].

To objectively assess the severity of pneumonia, several studies have focused on identifying independent predictors associated with adverse health outcomes. The pneumonia severity index (PSI) and CURB-65 scores are the most commonly recognized prediction tools for classifying patients with pneumonia in accordance with international guidelines [4, 8–10]. The PSI comprises 20 clinical and laboratory parameters, facilitating the classification of low-risk community-acquired pneumonia (CAP) patients into five distinct risk classes [8]. While the PSI exhibits robust discriminatory capacity in classifying patients into appropriate risk groups, its complex calculation poses a challenge for its clinical application, especially in the demanding settings of the emergency department (ED) [10–12]. In contrast, CURB-65 was developed from five readily measurable factors, rendering it user-friendly [4]. Several validation studies have indicated that its capability to predict mortality associated with CAP is nearly comparable to that of the PSI [6, 13–15]. However, both tools feature several variables with dichotomous and arbitrary cutoffs, limiting their predictive accuracy [16–19].

Machine learning (ML) is a widely explored domain in medicine presently. Innovative methodologies, including recursive partitioning, decision tree analysis, and random forest, provide a more robust approach to predicting clinical outcomes than traditional predictive models [20]. Various prediction models for pneumonia have been developed by training models with diverse clinical data sources, encompassing vital signs, medical history, laboratory tests, and even chest radiographs. Certain studies have demonstrated promising findings when comparing ML models with traditional prediction models [16, 20–23]. However, these complex input data mainly originated from various sources, necessitating multiple data point acquisition processes, additional blood draws, incurring costs, and proving challenging to uniformly and promptly obtain. Therefore, we developed a blood culture prediction model in our previous study, known as the blood culture prediction index (BCPI) model, exclusively leveraging complete blood counts (CBC) and differential leukocyte count (DC) data for bacteremia detection [24]. The BCPI model has demonstrated superior accuracy in predicting bacteremia compared to methods utilizing C-reactive protein (CRP) and procalcitonin (PCT) data. However, the applicability of the BCPI model in specific infections, such as pneumonia, remains unevaluated and unconfirmed.

Therefore, this study aimed to compare the performance of the BCPI model with the widely employed CURB-65 score in assessing pneumonia severity in an ED setting and their ability to predict prognosis following admission. Furthermore, we aim to construct a novel ML-based model by incorporating the CURB-65 score into the BCPI model and evaluate whether this approach enhances efficiency. Through these methods, we sought to extend the previously published BCPI model to pneumonia to generate additional value and provide potentially better predictive tools for pneumonia assessment.

## Methods

### Study setting

This retrospective study was conducted at Linkou Chang Gung Memorial Hospital (CGMH)—a 3400-bed tertiary medical center in Taiwan. The data utilized in the study were sourced from the Chang Gung Research Database (CGRD), which constitutes a de-identified duplicate of the clinical database at the hospital [25]. The study was approved by the Institutional Review Board of the hospital, and informed consent was waived (IRB No.: 202201120B0C101).

### Study population

**Fig. 1:**
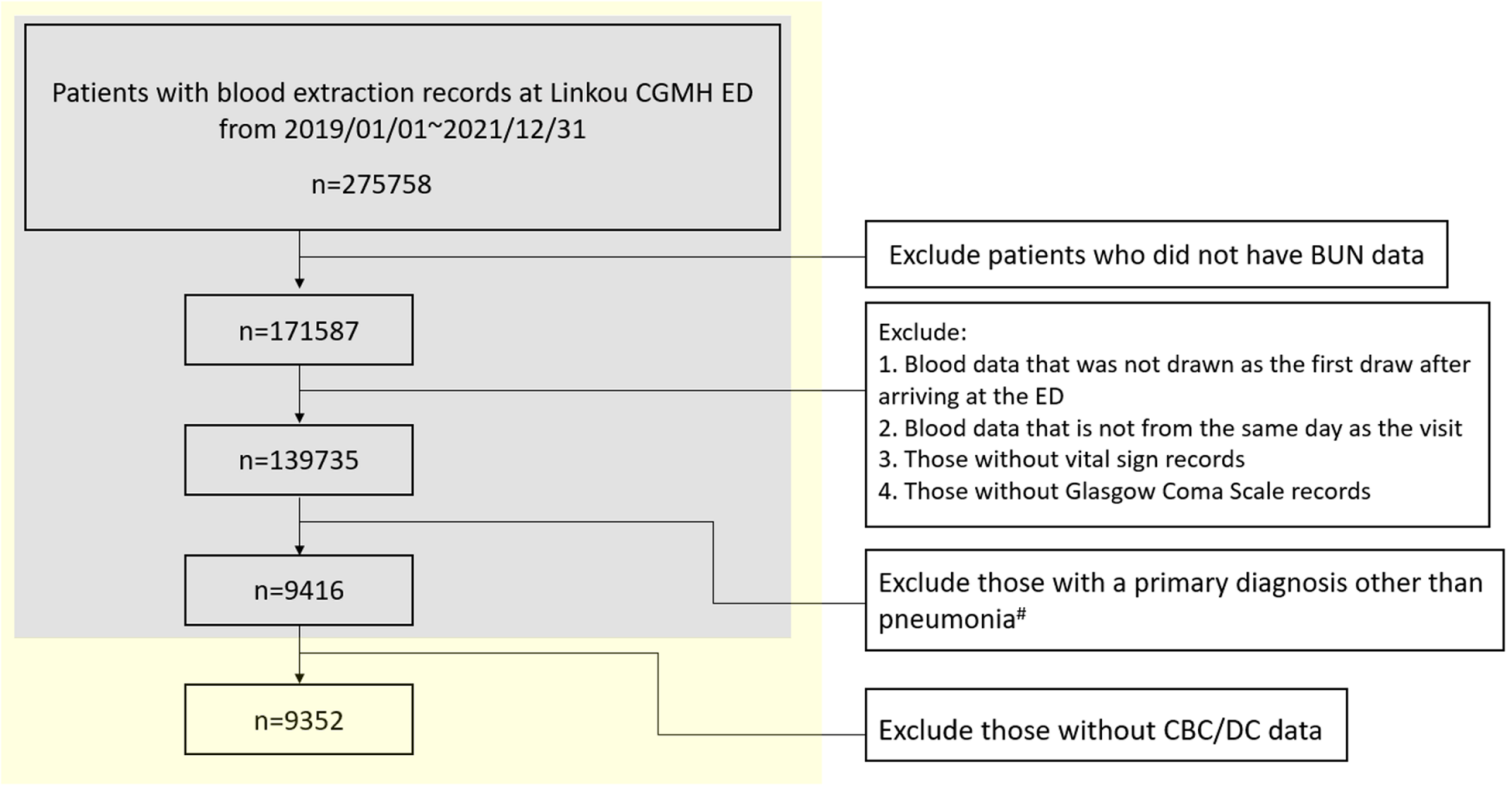
Research sample flowchart. The gray-shaded sections in the flowchart enable the calculation of the CURB-65 score for patients with pneumonia, while the yellow-shaded sections aid in computing the BCPI. ^#^ ICD: J10.0, J11.0, J12, J13, J14, J15, J16, J17, and J18

In our previous study, the BCPI model was trained using data from the same database from 2014—2018 [24]. In this study, data were extracted from patients who visited the ED between January 1, 2019, and December 31, 2021, with a primary pneumonia diagnosis. The inclusion comprised the following criteria: availability of CBC/DC and blood urea nitrogen (BUN) laboratory data on the same day of the ED visit, alongside data on age, sex, and assessment of respiratory rate, blood pressure, and consciousness level (Glasgow Coma Scale), conducted during triage on the day of the ED visit. These criteria ensure that the CURB-65 score and BCPI can be calculated and used to predict the prognosis for every included patient. Overall, 9,352 patients were enrolled in the analysis (Fig. 1).

### Training data preparation

The parameters used in the BCPI model mentioned above encompassed red blood cell count, hemoglobin, hematocrit, mean corpuscular volume, mean corpuscular hemoglobin, mean corpuscular hemoglobin concentration, red cell distribution width, nucleated red blood cell, white blood cell count, segmented neutrophil, band basophil, eosinophil, lymphocyte, atypical lymphocyte, plasma cell, plasmacytoid cell, hypersegmented cell, blast cell, myelocyte, meta-myelocyte, monocyte, promonocyte, and platelet count. The DC data were expressed as percentages.

To convert BUN, confusion, respiratory rate, blood pressure, and age into binary variables for model training and validation, we employed the cutoff values defined in the CURB-65 score (BUN level > 7 mmol/L, respiratory rate ≥ 30 breaths/min, systolic or diastolic pressure of < 90 mmHg and < 60 mmHg, respectively, and age ≥ 65 years old) [4]. Based on the original definition of CURB-65, "confusion" refers to a specific mental test or new disorientation of a person, place, or time [4, 26]. However, owing to retrospective research limitations, we could not confirm from the database whether patients were assessed for "confusion" based on the specified definition above. Therefore, we employed the Glasgow Coma Scale. Subsequently, we established a cutoff value of < 15. The limitations stemming from this approach are comprehensively discussed in the limitations within the discussion section.

### Developing prediction models

In this study, we assessed the effectiveness of risk stratification using the BCPI model compared to that of the CURB-65 score. Subsequently, the CURB-65 and BCPI models were integrated to create a new Cox regression model. Survival analysis was then conducted using in-hospital mortality as the endpoint. The model underwent training with data from patients hospitalized in 2019 and 2020, followed by validation using data from 2021. The significance of CRP as an important indicator in assessing pneumonia severity and prognosis has been highlighted in previous studies [27–29]. As a preliminary step, we explored substituting BCPI with CRP and integrating it with CURB-65 to develop a new model within our study cohort. Subsequently, the performance of this alternative model was assessed.

### Performance evaluation and statistical methods

To assess the prognostic performance of CURB-65 and compare it to that of the BCPI model for patients with pneumonia in the ED, we categorized patients into low- (CURB-65 score ≤ 1), moderate- (CURB-65 score = 2), and high-risk (CURB-65 score ≥ 3) groups based on their CURB-65 scores. This classification method aligns with the approach outlined in the 2009 British Thoracic Society (BTS) guidelines for managing CAP in adults [9]. We categorized the same group of patients with pneumonia into three risk groups based on the BCPI, ensuring that each group contained the same number of patients as those defined via the CURB-65 risk scores. Subsequently, we compared the 30-day all-cause mortality, admission, and in-hospital mortality rates for each risk group as defined via both assessment tools.

Following that, the Cox regression model was trained. It was then validated using data from hospitalized patients. We then compared the predictive performance of the Cox regression model and that of CURB-65 for in-hospital mortality, using the area under the receiver operating characteristic curve (AUROC) as the accuracy metric. The coefficients, *p*-values, and confidence intervals of each parameter in the Cox regression analysis were also provided. Regarding feature selection, we utilized Recursive Feature Elimination for the Cox regression model to iteratively eliminate features that exhibit the least effect on the AUC for mortality prediction [30]. We calculated detailed AUC values and corresponding confidence intervals for each model within our ablation study using the DeLong test [31]. All ML models and calculations, including mortality rate calculations, AUROC, and other metrics, were performed using Python 3.7 (https://www.python.org/). Statistical analyses involving variance and correlation coefficients were conducted using SPSS (IBM SPSS Statistics 19, Chicago, IL).

## Results

Patients who had CBC/DC and parameters included in the CURB-65 score collected and available during their pneumonia diagnosis in our ED were included in this study. Overall, 9,352 patients meeting this criterion were enrolled, with 6,655 hospitalized.

### Patient characteristics

Table 1 presents an overview of the primary information regarding the study participants. We classified all 9,352 patients into low- (CURB-65 score ≤ 1), medium- (CURB-65 score = 2), and high-risk (CURB-65 score ≥ 3) groups based on their CURB-65 scores, with 4,654, 2,513, and 2,185 patients in each group, respectively. The mean and median ages exhibited a gradual increase with rising risk levels, with males constituting the majority, making up approximately 60% of the patients across all risk groups. Positive finding proportions for each factor within CURB-65 also increased as the risk levels rose. As risk levels escalated, hospitalization rates naturally rose as well: 63% (low-risk), 78.7% (medium-risk), and 80.0% (high-risk). Similarly, 30-day mortality rates were consistent with this pattern: 5.4% (low-risk), 13.8% (medium-risk), and 26.8% (high-risk). Subsequently, an analysis was conducted on the 6,655 hospitalized patients, who were also divided into three risk groups based on their CURB-65 scores: low- (*n*=2,930), medium- (*n*=1,977), and high-risk (*n*=1,748) groups. The in-hospital mortality rates were calculated for each group, which were 7.9%, 15.1%, and 26.0%, respectively. To compare and validate the performance of the BCPI model to that of the CURB-65, patients were grouped into three risk categories, with an equal number of patients in each group based on their CURB-65 scores. Furthermore, we analyzed patient characteristics and calculated 30-day mortality for all emergency patients and in-hospital mortality for admitted patients. In this context, within the BCPI group, the proportions of positive findings for each CURB-65 factor still increased with increasing risk. However, compared to the CURB-65 cohort, distinct variations emerged. For instance, among those categorized as low-risk via CURB-65, only 26.1% were > 65 years of age, whereas the BCPI low-risk group exhibited a notably higher proportion at 47.0%. This discrepancy underscores how the BCPI model categorizes patients with pneumonia into risk tiers in a manner distinct from CURB-65. Nevertheless, the predictive ability of the two methods for the 30-day mortality rate among emergency patients and the in-hospital mortality rate among admitted patients were comparable across all risk categories (Table 2).

**Table 1:** Patient characteristics. We categorized the BCPI of the patients into three risk groups, each with an equal number of patients based on their CURB-65 scores, and then analyzed patient characteristics

**Methods and risk groups**	**All**	**CURB-65**			**BCPI model**		
		**low**	**medium**	**high**	**low**	**medium**	**high**
Number of cases	9352	4654	2513	2185	4654	2513	2185
AGE (mean)	63.0±26.0	47.6±26.6	75.5±15.0	81.3±10.2	54.2±30.0	70.9±18.3	72.6±16.1
Male (%)	62.5	60.0	66.2	63.7	59.3	62.8	69.2
Confusion (%)	28.2	4.5	30.0	76.8	18.9	35.0	40.4
Urea > 7 mmol/L (%)	45.0	12.7	64.4	91.3	29.2	55.4	66.6
RR ≥30 breaths/minute (%)	5.8	1.7	4.1	16.2	4.1	6.4	8.6
SBP < 90 mmHg or DBP < 60mmHg (%)	19.5	7.4	16.1	49.3	13.7	21.0	30.3
Age ≥ 65 years old (%)	58.4	26.1	85.4	95.9	47.0	69.2	70.1
CURB65 score (mean) (%)	1.6	0.5	2.0	3.3	1.1	1.9	2.2
CURB65 score (median) (%)	2	1	2	3	1	2	2

**Table 2:** Admission rate and mortality analysis: CURB-65 vs. BCPI

For all patients (*n*= 9,352)	CURB-65				BCPI model		
	Number of cases	Admission rate (%)	30-day mortality (%)		Number of cases	Admission rate (%)	30-day mortality (%)
Low-risk (CURB <= 1)	4654	63.0	5.4	Low-risk group	4654	64.7	6.2
Medium-risk (CURB = 2)	2513	78.7	13.8	Medium-risk group	2513	75.7	15.0
High-risk (CURB >=3)	2185	80.0	26.8	High-risk group	2185	79.7	23.6
For hospitalized patients (*n*=6655)	CURB-65				BCPI model		
	Number of cases	In-hospital mortality (%)	30-day mortality (%)		Number of cases	In-hospital mortality	30-day mortality (%)
Low-risk (CURB <= 1)	2930	7.9	7.1	Low-risk group	2930	7.4	7.0
Medium-risk (CURB = 2)	1977	15.1	14.0	Medium-risk group	1977	17.3	15.7
High-risk (CURB >=3)	1748	26.0	24.8	High-risk group	1748	24.5	23.1

### Cox regression model performance

In this study, a Cox regression model was constructed by integrating the BCPI model with the CURB-65 score. The training set comprised cases from the admission group between 2019 and 2020 (n=4,891), while the testing set comprised cases from 2021 (n=1,764). We evaluated the performance of the Cox model and compared it with that of the CURB-65 score. Table 3 shows the results of multivariate logistic regression of variables in the Cox regression model, including coefficients, p-values, hazard ratio, and confidence intervals. Our analysis revealed that all attributes significantly influenced the prediction, with "urea" making the greatest contribution, except for the continuous variable "BCPI."

**Table 3:** Multivariate logistic regression of variables in the Cox regression model for predicting in-hospital mortality

**Variable**	**Coefficient**	**SE, coef**	**HR (95% CI)**	***p*-values**
**CURB65-C**	0.74	0.07	2.1 (1.82–2.43)	<0.005
**CURB65-U**	0.83	0.08	2.29 (1.96–2.68)	<0.005
**CURB65-R**	0.72	0.1	2.04 (1.67–2.51)	<0.005
**CURB65-B**	0.29	0.08	1.33 (1.15–1.54)	<0.005
**CURB65-65**	0.39	0.09	1.48 (1.25–1.76)	<0.005
**BCPI**	3.17	0.28	23.84 (13.71–41.44)	<0.005

The prediction performance of in-hospital mortality rate among admitted patients was compared between the CURB-65 score and the Cox regression model (Table 4). As we utilized the Cox regression model to stratify patients into low-, medium-, and high-risk categories, with the same number of patients as in the CURB-65 score, no significant difference was observed between the two methods in predicting the prognosis of each risk group.

**Table 4:** In-hospital mortality analysis for the testing set in 2021: CURB-65 vs. Cox regression model

**For hospitalized patients (*n*=1764)**	**CURB-65**			**Cox regression model**		***p*-value**
	**Number of cases**	**In-hospital mortality (%)**		**Number of cases**	**In-hospital mortality (%)**	
Low risk (CURB <= 1)	645	8.1	Low risk	645	6.7	*p*=0.40
Medium risk (CURB = 2)	570	15.4	Medium risk	570	15.1	*p*=0.85
High risk (CURB >=3)	549	27.7	High risk	549	29.7	*p*=0.77

**Fig. 2:**
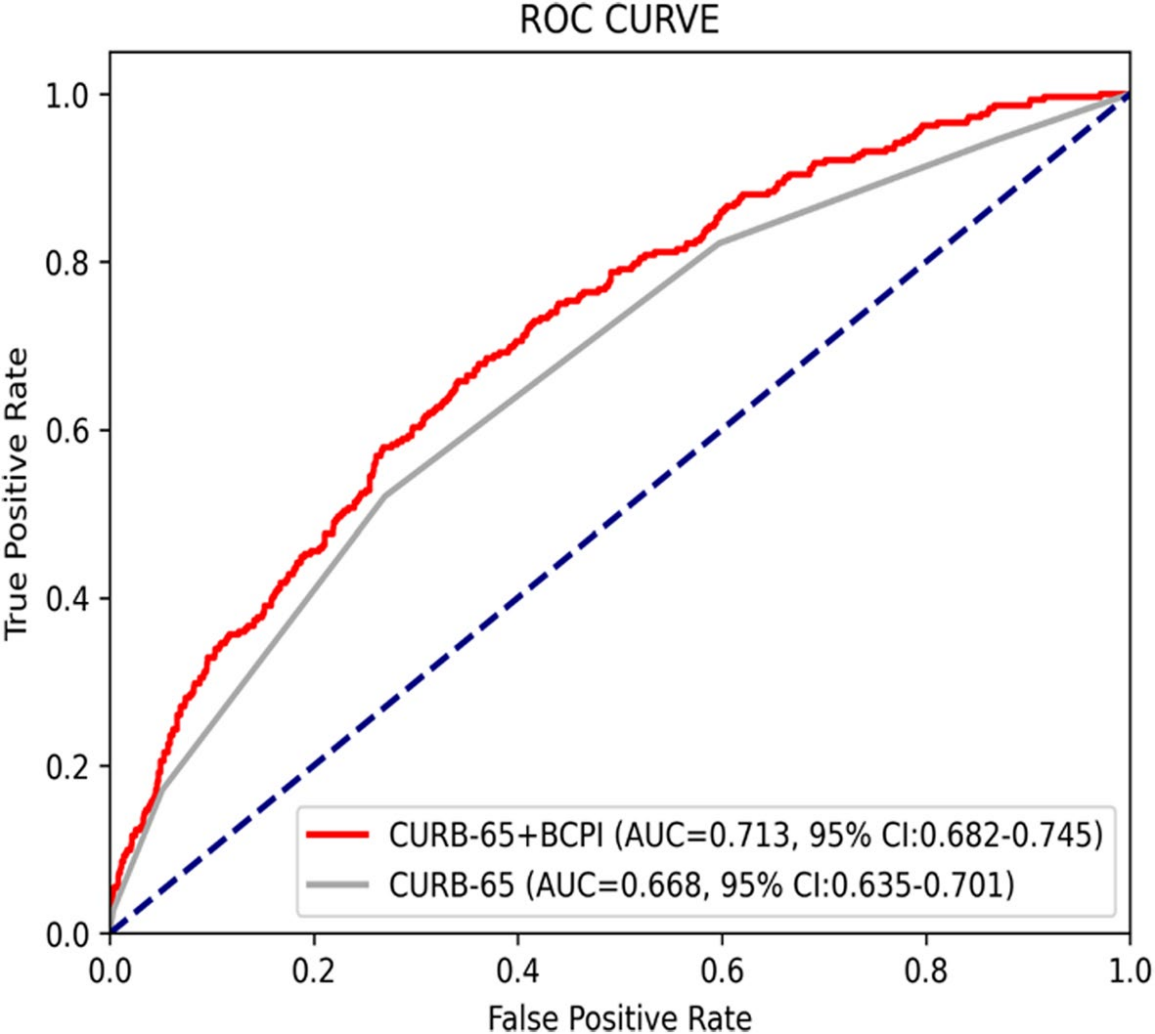
AUC for predicting in-hospital mortality rate (CURB 65 vs. Cox regression model)

The AUC for predicting in-hospital mortality rate was 0.668 for CURB-65, which increased to 0.713 for the Cox regression model, generally indicating acceptable discrimination for the Cox regression model (Fig. 2) [32]. The Cox regression model exhibited statistically significant discrimination in predicting in-hospital mortality within the lowest risk group (CURB-65=0) compared to the CURB-65 score (Table 5). The CURB-65 score specifically indicated a high mortality rate of 7.7%, whereas in the Cox regression model, it was 2.9% (*p*<0.001).

**Table 5:** Comparison of in-hospital mortality among hospitalized patients in 2021: CURB-65 vs. Cox regression model

**CURB-65 score**	**Number of cases**	**In-hospital mortality rate (%)**		***p*-value**
		**CURB-65**	**COX regression model**	
0	209	7.7	2.9	**<0.001**
1	436	8.3	8.7	0.61
2	570	15.4	14.4	0.47
3	423	24.1	25.8	0.44
4	113	37.2	40.7	0.44
5	13	61.5	76.9	0.19

### Ablation study using recursive feature elimination for the cox regression model

**Fig. 3:**
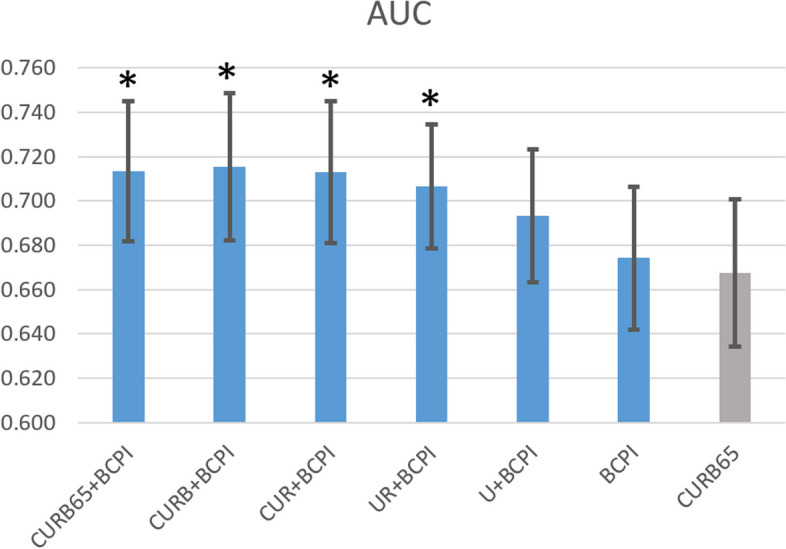
Ablation study using Recursive Feature Elimination for the Cox regression model. *Indicates statistical significance with p<0.05 compared to the AUC of CURB-65

Initially, Recursive Feature Elimination was performed with all features and iteratively removed the least important ones based on a ML algorithm's ranking. We observed that urea, the parameter contributing most significantly to the model after BCPI, was the last to be removed in the ablation study (Fig. 3). Furthermore, the complete Cox regression model showed a significant difference in predictive ability compared to CURB-65 (AUC 0.713 vs. 0.668, *p*<0.001). When only the BCPI parameter remained, the model exhibited comparable AUC values to CURB-65 (AUC 0.674 vs. 0.668, *p*=0.758), consistent with the results presented in Table 2. Furthermore, when the model included only UR+BCPI, the AUC surpassed 0.7, generally indicating acceptable discrimination, and demonstrated a significant difference in predictive ability compared to CURB-65 (AUC 0.707 vs. 0.668, *p*=0.015). Table 6 presents the detailed AUC values, confidence intervals, and *p*-values.

**Table 6:** AUCs, confidence Intervals, and *p*-Values for Recursive Feature Elimination ablation Study

**Model components**	**AUCs**	**95% CIs**	***p*-values**^**a**^
**CURB65+BCPI**	0.713	0.682-0.745	< 0.001
**CURB+BCPI**	0.715	0.682-0.747	< 0.001
**CUR+BCPI**	0.713	0.681-0.744	< 0.001
**UR+BCPI**	0.707	0.679-0.739	0.015
**U+BCPI**	0.693	0.663-0.724	0.138
**BCPI**	0.674	0.642-0.707	0.758
**CURB-65**^**b**^	0.668	0.635-0.701	-

^a^The p-values comparing the AUCs for each model within our ablation study with CURB-65

^b^The inclusion of CURB-65 in the last column of the table is for easy comparison and is not a result of the ablation study

## Discussion

Our study builds upon previous research findings on the BCPI model for bacteremia detection and evaluation, which relies solely on CBC/DC data. We found that this model also demonstrated promising results in predicting the likelihood of admission for ED patients with pneumonia, performing comparably to that of the widely used CURB-65 model. Furthermore, we constructed a new Cox regression model by integrating the covariates of the CURB-65 score and BCPI model. This model demonstrated superior mortality predictive capabilities than CURB-65 alone, particularly in discriminating low-risk patients. These findings suggest that the Cox regression model holds the potential as a valuable tool in emergency medicine for assessing pneumonia severity. Furthermore, BCPI may emerge as a significant component in developing future ML models for pneumonia.

Patients classified as low risk based on CURB-65 are suggested to be potential candidates for outpatient treatment [3, 4, 15, 33]. However, our data analysis revealed a relatively high admission rate of 63%, contrasting with previous recommendations. In addition, the 30-day mortality rate among the low-risk patients in our study was as high as 5.4%, which is concerning, contradicting our initial expectations. One possible reason for the high admission rate observed in our study could be attributed to Taiwan's National Health Insurance program. This program provides universal healthcare coverage to approximately 99% of the population, boosting one of the lowest administrative costs globally [34]. This results in greater accessibility to medical resources than in other countries. Additionally, we were unable to distinguish between community-acquired pneumonia (CAP) and hospital-acquired pneumonia (HAP) among patients in the ED. While HAP may represent a minority, it could still influence hospital admission and mortality rates. Furthermore, considering some patients classified as low-risk via CURB-65 may not be as safe as expected. Prior validation studies on the application of CURB-65 for CAP conducted by Aujesky et al. and Barlow et al. have revealed that a low-risk CURB-65 score of 1, conventionally deemed safe, correlates with a mortality rate of 3–4% [35, 36]. This finding is notably concerning as it indicates a substantially higher mortality rate than previously thought. Our study findings are consistent with these findings, suggesting that even among patients with uncomplicated CAP, CURB-65 may not effectively identify those at high risk of deterioration within the low-risk group, potentially resulting in underestimating the risk for patients. Additionally, previous studies based on CURB-65 consistently demonstrate that most low-risk patients presenting to the hospital are admitted, as observed in our study. This indicates a discrepancy between the recommendations derived from the CURB-65 score and the actual clinical decisions made [37, 38]. For example, Choudhury et al. found that out of 565 patients with low-risk CAP, 74.3% were admitted to the hospital [37]. Similarly, Aliberti et al. found that approximately 50% of patients who presented to the ED with a CURB-65 score of 0 or 1 were admitted based on clinical judgment rather than being treated as outpatients based on the score [38]. They identified hypoxemia and decompensated comorbidities as significant factors influencing this decision. Our study findings are consistent with these results, indicating that relying solely on CURB-65 for risk stratification in pneumonia may lead to an inaccurate assessment of the low-risk patients, potentially subjecting them to additional risk. The diagnostic and treatment guidelines for community-acquired pneumonia published by the American Thoracic Society (ATS) highlight a shortcoming of the CURB-65 score, particularly its limited ability to classify patients as low risk [10]. Thus, for patients with CAP with CURB-65 scores of 0 or 1, further assessments guided by clinical judgment or novel predictive models should be prioritized.

Compared to the CURB-65 score, our findings revealed that stratifying patients based on the BCPI did not yield a significant difference in the admission or 30-day mortality rates within each risk group. Presently, emergency physicians generally utilize the CURB-65 score as the primary basis for hospitalization decisions in clinical practice [6, 33, 35, 39]. However, the BCPI model, initially intended to predict bacteremia, exhibited comparable effectiveness for pneumonia in this context. Therefore, we posit that the efficacy of the BCPI model might surpass that of CURB-65. After integrating the CURB-65 and BCPI models, our Cox regression model demonstrated a higher AUC than that of the CURB-65 score. This novel assessment tool demonstrates effective discriminative capability, achieving an AUC of ≥ 0.7—a threshold generally recognized as indicative of "acceptable discrimination." In contrast, the AUC for the CURB-65 model alone, typically ranging from 0.5–0.7, is considered to reflect “poor discrimination” [32]. Additionally, the comparative analysis revealed a statistically significant improvement in predictive performance compared to the standalone CURB-65 model, with AUC values of 0.713 vs. 0.668, respectively (*p*<0.001) (Table 6). Upon validating the model using patient data from 2021, we discovered that the in-hospital mortality rate among inpatients with a CURB-65 score of 0 reached 7.7%. This highlights the challenges encountered by CURB-65 in accurately distinguishing low-risk patients, as discussed earlier. In contrast, the Cox regression model demonstrated an in-hospital mortality rate of only 2.4% in this particular group of patients, and the difference was statistically significant. While the remaining risk groups did not attain statistical significance, potentially owing to the limited sample size, the highest-risk group exhibited a clear trend toward enhanced discrimination. Therefore, the Cox regression model may serve as a more beneficial tool for identifying patients with low-risk pneumonia in future clinical practice. This can facilitate the safe discharge of patients who do not require hospitalization, thereby conserving medical resources. Additionally, it can aid in identifying patients at risk while ensuring they receive appropriate treatment.

Table 3 shows that all variables in the Cox regression model achieved statistical significance. Among them, "urea" emerged as the greatest contributor to the prediction, excluding the continuous variable "BCPI." In our previous BCPI model, we identified platelet count, monocyte percentage, lymphocyte percentage, segmented neutrophil percentage, and leukocyte count as the five significant features based on importance [24]. The identified factors align with those of other studies that highlight the significance of the neutrophil-to-lymphocyte ratio (NLR) in peripheral blood as a prognostic biomarker in infectious diseases, including pneumonia. NLR can also serve as a bacteremia predictor [40–42]. Additionally, the ablation study using Recursive Feature Elimination for the Cox regression model, as depicted in Fig. 3, revealed that urea was the last parameter eliminated, highlighting its predictive importance within the BCPI model. The comprehensive Cox regression model demonstrated improved predictive capability than that of CURB-65 alone (AUC=0.713 vs. 0.668, *p*<0.001), indicating the benefit of our approach. Furthermore, a simplified model comprising only urea, respiratory rate, and BCPI achieved an AUC exceeding 0.7, typically indicative of "acceptable discrimination," and notably outperformed CURB-65. These findings confirm the synergistic potential of integrating basic laboratory data with respiratory rate assessments and underscore the substantial enhancement in predictive accuracy achievable beyond the CURB-65 model. In previous studies on ML models for pneumonia, researchers have discussed the feature importance of clinical variables. However, these studies have lacked ablation studies, which would provide valuable insights into model complementarity and feature selection for future ML advancements in pneumonia [43, 44].

Furthermore, several studies have employed inflammatory markers, including CRP and PCT, to monitor patient response during infectious disease treatment or predict prognosis [45–50]. In pneumonia cases, CRP and PCT are often used alongside prediction tools such as the CURB-65 score to assess severity and inform antibiotic therapy decisions [27–29]. In our study cohort, CRP data were available for 6,086 patients (65.07%). However, when we substituted BCPI with CRP and integrated it with CURB-65 to create a new model, this modification did not enhance predictive performance (Figure S1). Furthermore, CRP alone exhibited relatively poor performance, which contradicts findings in previous studies supporting its utilization in predicting CAP severity [49, 51]. This suggests that training the model using CBC/DC data can achieve comparable performance to models incorporating inflammatory markers. Moreover, CRP and PCT require additional blood draws and testing costs. Conversely, CBC/DC is the most commonly performed laboratory test for patients with unidentified infections. They are cost-effective, requiring only a single blood draw, and have a short turnaround time of approximately 22 min in the laboratory with total automation [52]. Additionally, the BCPI utilized in constructing the Cox regression model is a continuous parameter. It can be used to categorize patients into different risk groups as needed, unlike the binary parameters of CURB-65. The continuous nature of the model enables arbitrary cutoffs to be applied based on clinical needs, potentially making it applicable to different regions and hospitals at varying levels. The parameters used to train the model are feasible and readily available in most EDs. Upon receiving laboratory data, we can derive risk assessment values from the Cox regression model. These values can then be employed to inform treatment decisions and the healthcare management, particularly those at low risk. This is crucial for emergency physicians. Additionally, BCPI holds potential for expansion into other fields, such as COVID-19 pneumonia or various infectious diseases. It can also serve as an adaptable component for future ML models.

Prominent medical practice guidelines, including those provided by respected organizations such as the British Thoracic Society (BTS) and National Institute for Health and Care Excellence (NICE), recommend using the CURB-65 score along with clinical judgment to inform treatment decisions for patients with CAP [9, 53]. Our study revealed that the Cox regression model outperformed CURB-65 and demonstrated a better ability to differentiate risk levels among patients, consequently ensuring safe and appropriate medical care. Moreover, the Cox regression model only relies on five easily obtainable parameters from CURB-65, alongside CBC/DC data, routinely tested for almost all ED patients. This significantly enhances the clinical usability of the Cox regression model.

In this study, we successfully developed a highly effective prediction model for pneumonia; however, we acknowledge that this study had some crucial limitations. First, the retrospective design of our study at a single center may introduce bias and confounding factors that might influence our results. Additionally, the generalizability of our findings to other healthcare settings could be affected by differences in patient populations across various centers. Second, owing to the retrospective research constraints, we could not confirm the presence of "confusion" in patients using the specific definition outlined in the CURB-65 criteria. Conversely, we used the GCS as an alternative for analysis. Although GCS may not perfectly align with the original concept of "confusion" in the CURB-65 criteria, the GCS variable continued to significantly contribute to the Cox regression analysis model. Furthermore, compared to clinical evaluations performed by emergency physicians, the GCS may provide a simple and more objective measure. This may better suit the demands of clinical practice, especially in busy emergency department settings. Moreover, although we endeavored to exclude cases of HAP or ventilator-associated pneumonia (VAP), it is important to acknowledge that the emergency department may receive referrals of pneumonia patients from other hospitals or nursing homes. These cases could potentially diverge from typical CAP cases. While we anticipate the proportion of such cases to be minimal, their inclusion in our study could still influence the results and conclusions drawn from our research on CAP. Finally, it is important to highlight that we only included patients with pneumonia who underwent a BUN test for CURB-65 calculation. However, the frequency of BUN testing is not as high as that of CBC/DC tests (over 99%). Consequently, excluding patients who did not undergo this test may introduce potential bias into our analysis. Therefore, more studies may be necessary to validate the performance of this ML approach further. Future investigations should prioritize initiating pilot trials to assess the effectiveness of the proposed analytical methods in enhancing clinical prognostic decision-making. Essential to this effort will be establishing partnerships with medical professionals to ensure the models are appropriately integrated into clinical workflows. These studies will seek to validate the effectiveness of these approaches in real-world settings and enrich the discussion on integrating algorithmic models into healthcare decision-making, a crucial advancement in patient care. However, despite these limitations, it is essential to emphasize the significant sample size of our study, comprising 9,352 individuals meeting the inclusion criteria. This sample size is significantly larger than those of previous studies, whether for validating prediction models or developing new machine-learning-based models. It provides robustness and statistical power, enhancing the reliability and generalizability of our study results.

## Conclusions

This study demonstrated that the BCPI model, constructed using CBC/DC data and ML techniques, performs comparably to the widely used CURB-65 in predicting outcomes for patients with pneumonia in the emergency department. Furthermore, by incorporating the CURB-65 score with the BCPI model into a Cox regression model, we have demonstrated enhanced prediction capabilities, particularly for low-risk patients. Given its simple parameters and straightforward training process, the Cox regression model holds promise as a more effective prediction tool for categorizing patients with pneumonia in the emergency room.

### Supplementary Information

**Supplementary Material 1.**

## Data Availability

The data used in this study, sourced from the Chang Gung Research Database at Chang Gung Memorial Hospital, are not publicly accessible owing to usage limitations and licensing agreements. However, individuals interested in accessing the data can request it directly from the authors of the study, provided they obtain the necessary permissions from the Chang Gung Research Database and Chang Gung Memorial Hospital.

## Abbreviations

ML: Machine learning
BCPI: Blood culture prediction index
CBC: Complete blood count
DC: Differential leukocyte count
ED: Emergency department
AUC: Area under curve
ROC: Receiver operating characteristics
PSI: Pneumonia severity index
CAP: Community-acquired pneumonia
HAP: Hospital-acquired pneumonia
VAP: Ventilator-associated pneumonia
CRP: C-reactive protein
PCT: Procalcitonin
CGMH: Chang Gung Memorial Hospital
CGRD: Chang Gung Research Database
BUN: Blood urea nitrogen
GCS: Glasgow Coma Scale
ATS: American Thoracic Society
BTS: British Thoracic Society
NICE: National Institute for Health and Care Excellence
NLR: Neutrophil-to-lymphocyte ratio
