# Supplementary material for: Enhancing pneumonia prognosis in the emergency department: a novel machine learning approach using complete blood count and differential leukocyte count combined with CURB-65 score
Source: BMC Med Inform Decis Mak. 2024 May 3;24:118. doi: 10.1186/s12911-024-02523-1 (PMC11069213; doi:10.1186/s12911-024-02523-1)
Supplement: Supplementary file 1 — Supplementary Material 1. [file 12911_2024_2523_MOESM1_ESM.docx]

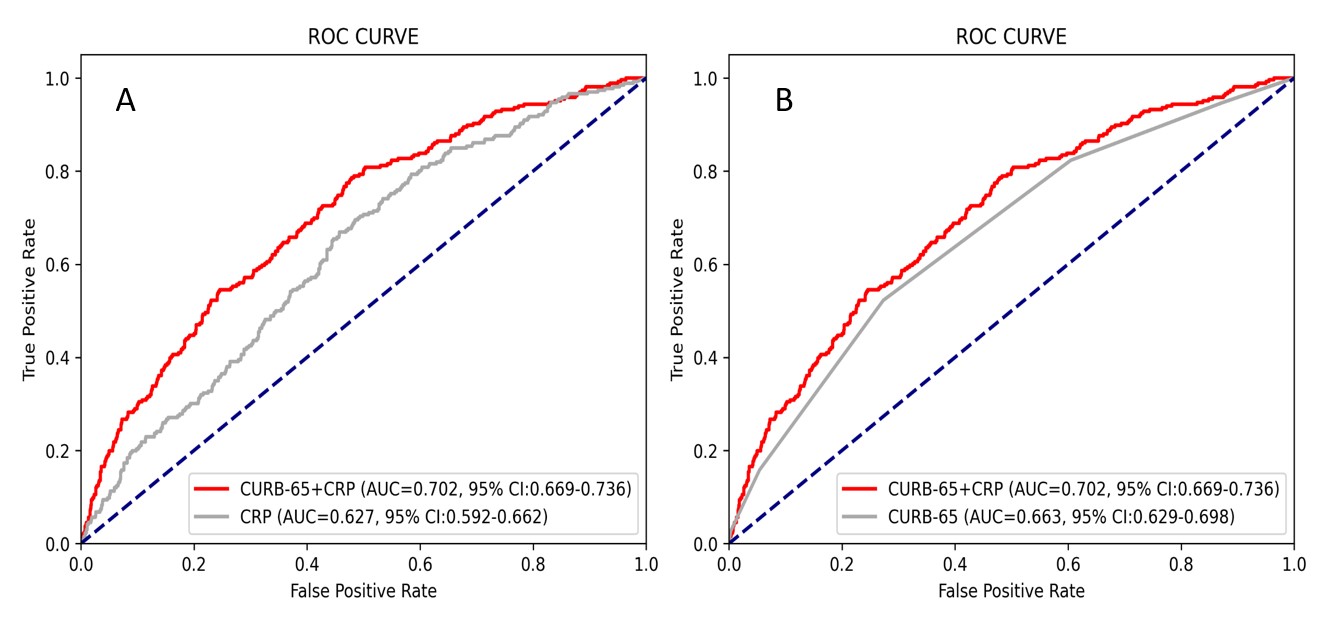
**Additional file 1: Figure S1. AUC for predicting in-hospital mortality rate: CURB 65 vs. CURB-65 + CRP**

A: CURB-65 + CRP vs. CRP alone

B: CURB-65 + CRP vs. CURB-65 alone
